# Adenocarcinoma mimicking appendicular lump: a diagnostic dilemma—a case report

**DOI:** 10.1186/s12957-016-1036-9

**Published:** 2016-11-11

**Authors:** Sanket Kalpande, Jayashri Pandya, Tushar Sharma

**Affiliations:** Department of General Surgery, TNMC & BYL Nair Ch Hospital, Mumbai, Maharashtra India

**Keywords:** Adenocarcinoma, Appendix, Iliac fossa lump

## Abstract

**Background:**

Primary appendiceal adenocarcinoma is a rare tumor, mucinous variety being common. This case is reported to highlight the unusual presentation and diagnostic difficulty of appendiceal adenocarcinoma.

**Case presentation:**

Patient presented with acute appendicitis with ill-defined tender lump which responded to conservative management.

**Conclusions:**

High index of suspicion should be kept in mind for elderly patients presenting with appendicular lump. Every effort should be made during elective appendectomy to remove stump in case of sloughed out appendix.

## Background

Primary appendiceal adenocarcinoma is a rare entity frequently discovered by pathologist after appendectomy. Patients commonly present as acute appendicitis or may present as a palpable mass in the right iliac fossa [1]. Primary appendiceal adenocarcinoma accounts for 0.05 to 0.2% of all appendectomies and only 6% of all malignant tumors of appendix [2]. This patient presented as an appendicular lump which resolved on conservative management. Later on, after appendectomy, histopathology of appendicular stump was suggestive of mucinous adenocarcinoma. Right radical hemicolectomy was performed.

## Case presentation

A 55-year-old female presented with recurrent abdominal pain in the peri-umbilical region and right iliac fossa of 6-month duration which had increased since 5 days, associated with one episode of non-bilious vomiting. There was no history of fever, altered bowel or bladder habit or loss of appetite. On examination, patient was febrile with pulse of 104/min. Systemic examination was normal. Abdominal examination revealed an ill-defined tender lump in the right iliac fossa with no guarding and rigidity. Rectal examination was unremarkable. Investigations revealed white blood cell (WBC) count of 13,800/mm^3^. The rest of the investigations were normal.

Ultrasonography of the abdomen showed thickened loops of ileum and caecal wall suggestive of inflammatory etiology. In view of ill-defined lump, possibility of appendicular abscess or ileo-caecal Koch’s was considered. Erythrocyte sedimentation rate (ESR) was 22 mm (0–20 at the end of 1 h), and serum adenosine deaminase (ADA) level was 48.8 IU/L (0–40 IU/L) which were within normal limits.

**Fig. 1:**
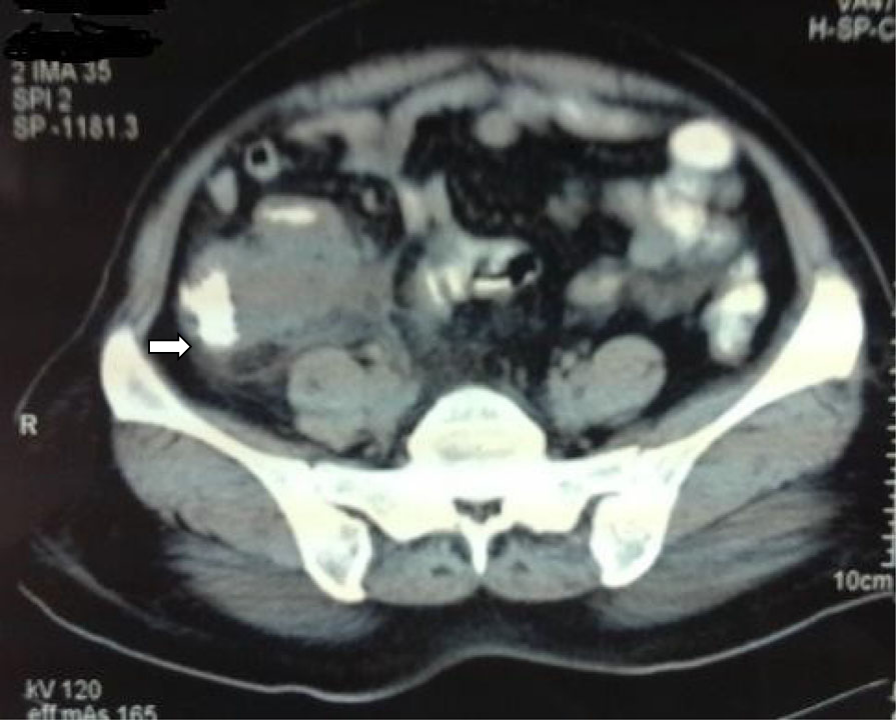
CT scan showing appendicular lump. *Arrow* showed an ill-defined hypo-dense peripherally enhancing collection about 4 × 3.65 cm in the right iliac fossa. Few air locules with asymmetric mural thickening of ileum, caecum, and ileocaecal junction

**Fig. 2:**
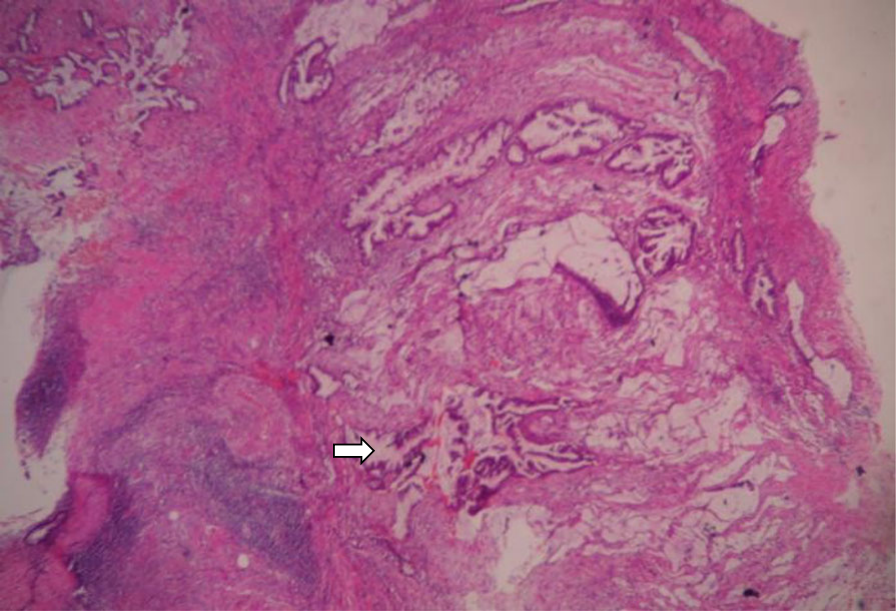
HP slide showing mucinous adenocarcinoma. H&E stain (×40). Section showing wall of appendix with prominent lymphoid follicles and tumor tissue adjacent to it containing tall cells with high nucleocytoplasmic ratio (*arrow*), arranged in glandular pattern with extracellular mucin

Computerized tomography (CT) scan of abdomen (Fig. 1) suggested possibility of sealed appendicular perforation or appendicular lump of inflammatory etiology. The appendix was not separately visualized. Patient was started on conservative management and had an uneventful recovery. The lump gradually resolved. Elective appendectomy was planned after 6 weeks. At elective appendectomy, the appendix appeared sloughed off with a short stump. Appendectomy was performed, and specimen was sent for histopathology. There were no lymphadenopathy and no ascites, and tubes and ovaries were normal. Histopathology (Fig. 2) revealed moderately differentiated mucin secreting adenocarcinoma of the appendix. As sloughed out stump was sent for histopathology, negative margins could not be confirmed.

Post appendectomy, CT abdomen did not show any mass or evidence of metastasis. Serum carcino embryonic antigen (CEA) levels were 2.02 ng/ml (0–5 ng/ml). Right radical hemicolectomy with regional lymphadenectomy was performed after 2 weeks of appendectomy after histopathology report was available. Postoperative course was uneventful. Histopathology revealed moderately differentiated mucin secreting adenocarcinoma. Resection margins were negative, and all 12 lymph nodes resected from the specimen were negative for tumor. The tumor was staged as T_4_N_0_M_0_ (Modified Astler Coller B3). Multidrug adjuvant chemotherapy of 5-fluorouracil, leucovorin, and oxaliplatin (FOLFOX) was given. Radiotherapy has been given for 15 cycles. Patient was asymptomatic, and CEA levels at the end of 12 months were normal. CT scan at the end of 12 months revealed no evidence of recurrence.

## Conclusions

The emphasis of the case report is on highlighting the difficulty in preoperative diagnosis of appendicular carcinoma in patients presenting with appendicular lump and sepsis. In elderly patients presenting with appendicular lump, the differential diagnosis of malignancy should be kept in mind. During interval appendectomy, even if sloughed off appendix, specimen should be sent for histopathology. Conformation of carcinoma even if rare would save patient from dissemination of malignancy due to violation of planes at the time of surgery and better adherence to oncologic principles during the single-stage procedure as opposed to a two staged one.

### Discussion

The tumors of the appendix are of three histological types: carcinoid tumors (88%), cystic adenocarcinoma (8%), and primary adenocarcinoma (4%) [3]. Primary adenocarcinoma of appendix is a rare neoplasm accounting for 0.05 to 0.2% of all appendectomies. Primary adenocarcinoma of appendix is usually classified according to histopathological examination into four distinct subtypes with varying frequencies: colonic type adenocarcinoma, mucinous adenocarcinoma, signet ring-type adenocarcinoma, and others [4]. Of all the variants, mucinous histology is a common variant and has the best prognostic factor [5]. Most mucinous types are well differentiated and slow growing [6]. Adenocarcinoma of appendix spreads via local invasion, lymphatic vessels, and blood stream. These tumors usually present as acute appendicitis, chronic recurrent appendicitis, appendicular abscess or rarely appendicular lump or as primary bladder cancer, hydronephrosis or pelvic mass. There are no symptoms specific to appendiceal cancer.

Malignancies of appendix are rarely diagnosed preoperatively or intraoperatively. Most tumors are identified only after histopathological examination of resected specimen following appendectomy or as an incidental finding during exploratory laparotomy for other surgeries [6]. While simple appendectomy seems to be sufficient for pT1 carcinoma of appendix, most tumors present as advanced invasive carcinomas and secondary right radical hemicolectomy is usually recommended as the operative treatment of choice [7, 8]. Malignant infiltration of loco regional lymph nodes is expected in about one third of the patients especially in advanced invasive tumors. Adjuvant chemotherapy is recommended [4, 9].

In cases of adenocarcinoma of appendix with intra-abdominal metastasis, the treatment consists of aggressive debulking followed by adjuvant chemo-radiotherapy [9–11]. The prognostic factors in primary adenocarcinoma of appendix depend upon tumor stage, histology, grade, size, and the type of surgery [12]. Patients with appendiceal adenocarcinoma undergoing right radical hemicolectomy have better survival as compared to appendectomy alone [1].

## Abbreviations

ADA: Adenosine deaminase
CEA: Carcino embryonic antigen
CT: Computerized tomography
ESR: Erythrocyte sedimentation rate
WBC: White blood cell
